# "The Hospital" Nursing Mirror

**Published:** 1897-05-22

**Authors:** 


"The Hospital" Nursing Mirror.

Being the Nursing Section of "The Hospital."

[Contributions for this Section of "The Hospital" should be addressed to the Editor, The Hospital, 28 & 29, Southampton Street, Strand, London, W.C., and should have the word "Nursing" plainly written in left-hand top corner of the envelope.]

### News from the Nursing World.

### PRINCESS CHRISTIAN AND THE ARMY NURSING RESERVE.

On Wednesday afternoon H.R.H. Princess Christian, who was accompanied by Princess Victoria of Schleswig-Holstein, presented certificates of appointment and badges to fifty nurses of the Army Nursing Reserve. The ceremony took place at the United Services Institution, Whitehall, the Marquis of Lansdowne, Secretary of State for War, presiding. Lady Lansdowne, Lord Loch, Dr. Bezly Thorne, Miss Wedgwood, and many other visitors were present. Lord Lansdowne, in an opening speech, explained the purport of the Army Nursing Reserve, full particulars of which have already been given in The Hospital. It is to consist of 100 nurses, of whom 65 have already been enrolled. The medals are of silver, and round them are engraved the words, "Princess Christian's Army Nursing Reserve."

### THE COUNTESS CADOGAN AT AN IRISH HOSPITAL.

Last month a state visit was paid by the Countess Cadogan to the National Hospital for Consumption for Ireland, at Newcastle, co. Wicklow. Lady Cadogan was received by Mr. R. O'B. Furlong (chairman of the board of governors), Miss Powell (the lady superintendent), and Dr. Steede (resident medical officer). The High Sheriff of the County, Sir Henry Cochrane, and a number of visitors were also present. Lady Cadogan expressed herself as much pleased with all the arrangements made for the comfort of the patients, and the bright and cheerful appearance of the bed-rooms and sitting-rooms (the hospital is built on the separate system), making minute inquiries into the details of management and treatment. She was also much interested in the extensive ventilating and heating apparatus by which fresh warmed air is forced through the building, and the atmosphere kept pure and sweet.

### DR. JOSEPH BELL ON THE PROFESSIONAL TRAINED NURSE.

Dr. Joseph Bell's excellent remarks "On the relation of the Trained Nurse to the Profession and the Public," which appeared in the March and April numbers of *The Scottish Medical and Surgical Journal*, have now been reprinted in pamphlet form, and every nurse should read them if she gets the chance. Dr. Bell enters with full appreciation into the mutual difficulties of nurses and the public, judging that in the main the present unpopularity of the trained nurse is due not so much to herself as to the discredit brought upon her by untrained masqueraders in uniform, but at the same time admitting that "airs and graces, meals at all odd hours . . . . constant ringing of bells" will sometimes cause a nurse in a middle-class house with few servants to be "an intolerable burden." Another point touched upon is the so-called "trained nursing" introduced into workhouse infirmaries by economical boards of guardians, whose idea of re-organisation is to get one trained nurse for perhaps 100 bed-rid pauper patients, and to ask her to nurse them day and night with the aid of three or four semi-idiotic, possibly epileptic, young women. Then trained nursing bears the blame if one of these "boils a baby or poisons an old woman."

### THE NURSES' HOSTEL.

The Nurses' Hostel, in Percy Street, is, we understand, about to be formed into a limited liability company. Miss C. J. Wood hopes that eventually the property will be entirely owned and managed by the nurses. A new hostel is being built in Francis Street, Tottenham Court Road, planned with every regard to comfort, and will, it is expected, be ready for occupation in June.

### GODALMING AND THE NURSING SCHEME.

Several schemes were brought forward at a recent meeting at Godalming for commemorating the "record reign," but that of endowing and extending the present nursing institution was first favourite, and was carried by a large majority. The proposal includes the building of a home for the nurses and increasing the staff, and a strong committee has been formed to carry the project into execution.

### NEWS FROM THE EAST.

It is stated that a fully-equipped ambulance of five hundred beds, with surgeons and nurses, has been sent from Russia to the assistance of the Turkish Army. Reports from correspondents in the Turkish lines show that the field hospitals and the medical arrangements generally are excellent.—Somewhat late in the day the English Red Cross Society has now offered surgical appliances and money to the Porte, an offer which has been accepted.—The *Daily Chronicle* nurses are reported to be working in the hospitals which have been established at Chalcis and at Athens. The Queen of Greece, with the Crown Princess and Princess Marie, paid a surprise visit to the English hospital at Athens the other day, the Queen singling out for special notice an English volunteer, Captain Birch, who was wounded at Pharsala.

### NURSING IN AMERICA.

The New York nursing world has been lately exercised over a suggestion for the formation of a "protective association," discussed at meetings held in March and April. Mrs. O. Grafstrom was responsible for a paper setting forth the scheme, proposing the establishment of an organisation to be called "The Trained Nurses' Mutual Protective Association," which would aim at limiting, by Act of Legislature, the practice of nursing to nurses who have had two years' training in a hospital with 75 beds or more. It would also restrict "the practice of electro, mechano, and hydro therapy" to members of the medical profession and trained nurses, and would found a home and hospital for nurses. The proposal was opposed by Miss L. L. Dock, secretary of the American Society of Superintendents of Training Schools, on the grounds that "one of the greatest evils in the nursing profession was the training schools in connection with small hospitals," and that the suggested scheme would tend to lower rather than raise the standard of nursing. The majority of the nurses present at the various meetings were in favour of the idea, and ultimately officers were elected; and at a meeting on April 12th, at which some seventy graduate nurses were present, the draft of a Bill was read, discussed, and finally endorsed for presentation to the New York Legislature.

### QUEEN'S NURSES AND "TRUTH."

The pages of *Truth* have lately been opened to a discussion on the Queen's Jubilee Institute for Nurses, in the course of which the question of pay has come up. On this point "a lady conversant with the facts" has been good enough to write at some length, showing unconsciously and very conclusively the immense importance to nurses of the standard of pay which the Institute endeavours to maintain. She instances a case where a parish nurse was required for an inclusive salary of £50. A Queen's nurse who applied expected payment on a scale which would mean "an annual outlay of over £70," and the writer actually boasts that finally an independent nurse was engaged "whose terms were more reasonable," or in other words who allowed herself to be underpaid. There are many excellent and charitable people who in their desire to get skilled nursing for their poor at the lowest cost overlook the fact that the "labourer is worthy of his hire," and expect from the unfortunate nurse entire submission to be overworked and underpaid at their sweet will and pleasure. A district nurse cannot possibly be properly fed, housed, and clothed for an inclusive sum of £50 a year, and even if that sum would just cover bare expenses, how is she to save for her own old age on a bare pittance such as that? It is not a "living wage." The Institute is doing nurses an incalculable service by insisting upon the adequate payment of its nurses, and it is certain that that standard will be the generally accepted standard for district nurses in the future.

### PRINCE ALFRED HOSPITAL AND ITS MATRON.

Commenting on the nursing department of the Prince Alfred Hospital, Sydney, in the annual report of that institution, the directors remark that "the Matron has continued to fulfil the onerous duties of her post with marked interest, ability, and energy, to the great advantage of the institution; the high degree of efficiency and discipline maintained amongst the nursing staff is mainly the result of her influence and work." Miss McGahey's old friends in England will rejoice at this cordial appreciation of the excellent work she has done at the Prince Alfred Hospital.

### PORTADOWN DISTRICT NURSING SOCIETY.

At a public meeting on behalf of the Portadown District Nursing Society the highest praise was given to the work done by Nurse Camp since she has taken up her abode in the town. The speakers one and all united in acknowledging the need for the society, and the incalculable blessing which a trained nurse had proved herself amongst the poor. Dr. Hadden said that in the last few months he could mention several cases where, humanly speaking, the patients could not have recovered but for the care and attention of their "Queen's Nurse." Resolutions urging the claim of the society on the people of Portadown were passed.

### WEDNESBURY AND THE JUBILEE.

It is proposed in Wednesbury to celebrate the present year by the establishment of a district nurses' institute. A cottage hospital was the first suggestion, but to this idea various objections were raised, and it was abandoned. The plan now is to build a home in some suitable and central position at a cost of about £5,000, providing accommodation for about three nurses. It is estimated that the maintenance will be £250 or £300 a year, and it is hoped to raise a further sum of £5,500 to form the nucleus for endowment. If the scheme meets with sufficiently encouraging support the foundation stone of the new building will perhaps be laid by the Mayor on June 22nd.

### HOSPITAL BADGES AND MEDALS.

The proprietors of the Scientific Press will be glad to receive specimens of badges and nursing medals from the matrons of all hospitals and institutions throughout the country which possess these marks of distinction, to form part of their exhibit at the Victorian Era Exhibition, to be opened next week at Earl's Court. Every care will be taken of the specimens, which will be returned at the close of the exhibition, and matrons sending these interesting contributions are requested to affix a descriptive card to each badge or medal.

### THE EDITOR OF "THE HOSPITAL."

In answer to many kind inquiries from nurses, we are glad to be able to say that the Editor of The Hospital has returned from his travels very much improved in health. He desires to take this opportunity of thanking all those who have so kindly sent inquiries and sympathy, including the readers of The Hospital and the members of the Royal National Pension Fund for Nurses, through the secretary of which institution we have received repeated messages from nurses.

### SHORT ITEMS.

The annual meeting of the National British Women's Temperance Association is to take place at the Queen's Hall on Wednesday, June 2nd. The Lady Battersea has promised to preside, and Lady Henry Somerset and Canon Wilberforce will be among the speakers.—It is proposed to start a Queen's Nurse at Stonehaven this year, and Mr. Sydney Herbert, who is a constant summer visitor to the town, has offered a contribution of £50 towards the initial expenses and a yearly subscription of five guineas towards the nurse's maintenance.—The committee of the Northern Hospital, Liverpool, have arranged to give their nursing staff an extra week's holiday this Jubilee year. The annual leave is four weeks, and each member of the staff will this year have five weeks' holiday.—The annual meeting of the Wotton-under-Edge District Nursing Association was held on May 6th, at which an address was given by Miss Whitmore Jones upon the needs and work of cottage hospitals.—The parish of St. Augustine's, Croydon, is proposing to carry out a Jubilee scheme of its own, and a district nurse is to be established there forthwith. A very successful concert was held the other day in the parish hall on behalf of this good object. —A series of portraits of Queen's nurses is being exhibited at the Crystal Palace and Earl's Court Victorian Exhibitions by Messrs. Speaight, photographers, Regent Street.

### Pharmacy and Dispensing for Nurses.

By C. J. S Thompson.

### X.—DRAUGHTS—DROPS—EMULSIONS.

The draught is the name applied to a liquid medicine, the whole of which is to be taken for a dose. During the early part of this century the draught was the most popular method of administering medicine in liquid form, it being customary with medical practitioners to prescribe each dose in a separate bottle. When two doses are dispensed in one bottle a strip of paper should be affixed, showing an accurate division of the liquid. Draughts may vary in quantity from half to two ounces. They are prepared in a similar manner to mixtures, viz., first dissolving the solids in the liquid menstruum, then adding the fluids; or, if the solid substances are soluble in water, they should be stirred in a mortar with a little mucilage in order to suspend them, then placed in the bottle and labelled with directions to shake.

The term "drops" is usually applied to a medicine in concentrated form, the dose of which is ordered to be taken in drops, to be diluted with water by the patient. They are usually composed of a tincture, or a simple mixture of tinctures, and rarely give the dispenser trouble.

"Drops" are also frequently ordered for application to the eyes or ears. The former require considerable care in preparation, especially when minute quantities of alkaloids are ordered. When the quantity is too small to be weighed in the ordinary dispensing scales, a solution of definite strength should be made, from which an exact equivalent of the weight may be measured off. Solutions containing nitrate of silver and eserine should be sent out in coloured glass bottles to prevent the action of the light, and distilled water must always be used. Drops should be dispensed in glass-stoppered bottles, and the stopper capped with skin or parchment paper.

An emulsion is the name given to a mixture of an oil, fat, or resin, with water, it being rendered more or less permanent by the aid of another body such as a gum or an alkali, which medium is called the emulsifying agent. Milk may be taken as an example of an almost perfect natural emulsion.

If we half fill a bottle with olive oil, then add an equal quantity of water, and shake them together vigorously for a few minutes, the oil for a time will appear to have mixed with the water, forming an opaque mixture; allow it to stand for a short period and it will soon separate into two distinct liquids again. But if a small quantity of liquid ammonia be mixed with the oil before the water, and then the latter added gradually, and the bottle well shaken, a milky liquid will result, which will not readily separate on standing. In this case the ammonia acts as the emulsifying agent or medium, in breaking up the oil globules and so rendering them miscible with water. A perfect emulsion should be of a creamy consistence, exhibit no oil globules, and not separate on standing. Some oils emulsify with one agent better than another. One will form a better emulsion with mucilage than with an alkali, for another, yolk of egg will be found the best medium; therefore the dispenser must note from practical experience the right agent to employ in order to get the best result.

In making an emulsion a great deal depends on manipulative skill, and the manner in which the operation is conducted. While some oils simply require to be shaken up in a bottle with the emulsifying agent, others need careful trituration or stirring in a mortar.

The former and simplest method is usually adopted when alkalies, such as solutions of potash or lime, or tinctures of quillaia or senega, are employed as emulsifying agents. As an example of this class we may take the following process, which should be adopted for emulsifying balsam of copaiba.

The bottle should first be half filled with water, then two or three drachms of solution of potash added. The copaiba should then be poured in, without being allowed to touch the neck or sides of the bottle; then shake the liquids together vigorously until they assume a creamy consistence. The remainder of the water may then be added in small quantities at a time, the bottle being well shaken after each addition.

Cod liver oil may be emulsified with tincture of quillaia and lime-water in a similar manner. One drachm of the tincture is sufficient to emulsify two ounces of oil. Fill the bottle half full of water, to which add the tincture, and mix. Then pour in an ounce of the oil and shake well until the oil globules are broken up; add another portion and repeat the agitation, and so on, until all the oil is emulsified.

Where mucilage, powdered gum acacia, tragacanth, Irish moss mucilage, or yolk of egg are used as emulsifying media; trituration in a mortar is necessary. With this method the pestle requires skilful handling. The motion should be quick, light, and regular, care being exercised to stir in one direction only, and not to reverse it during the process. It is well to remember that in most cases the body to be emulsified must be added to the agent in small quantities at a time, each portion being thoroughly mixed before the next is added.

Castor oil may be emulsified with gum acacia in the following way: To one part of the powdered gum in a mortar, add gradually two parts of castor oil, and triturate well with the pestle until thoroughly mixed, then add all at once two ounces of water, and again triturate till the oil is completely emulsified. Flavouring may now be added, and more water if required.

Cod liver oil forms an excellent emulsion with powdered gum acacia if carried out in the following manner: Place six drachms of finely powdered gum acacia in a mortar, and add to it, while stirring constantly, three ounces of cod liver oil, which has been previously flavoured with a suitable essential oil, such as almonds or cinnamon. Ten drachms of water may next be added, the mixture being stirred quickly but lightly until it has a creamy appearance. The remainder of the water may now be added gradually, and any other liquid it is required to mix with it, the whole to form a product measuring six ounces.

A good emulsion of cod liver oil may also be made with Irish moss. Resinous bodies are usually emulsified with powdered gum acacia.

Balsam of copaiba may also be emulsified with powdered gum acacia, solution of potash, or yolk of egg. Sweet oil of almonds with solution of potash or ammonia. Spermaceti, or other solid fats, with yolk of egg. Balsam of Peru and tincture of benzoin with yolk of egg. Oil of male fern with mucilage, powdered acacia, or tincture of quillaia. The admixture of glycerine or spirit is usually fatal to the formation of a good emulsion. Turpentine may be emulsified with yolk of egg or soap. To prepare the yolk, the egg shell should be fractured about the centre by means of a sharp blow with a knife. Allow all the albumen to escape, then place the yolk in a mortar, break, and stir it well, and add the turpentine to it in small quantities, stirring constantly until they are thoroughly incorporated. When soap is used as the emulsifying agent, it should be placed in a mortar, and the turpentine added to it gradually, in small quantities, with rapid trituration. Each of the methods we have mentioned should be tried and practised by the student repeatedly, until the art of making a good emulsion is acquired.

### Post-Graduate Clinics for Nurses.

By a Trained Nurse.

### XIV.—SOME TEXT BOOKS AND TRADITIONS.

I remember how, in my probationer days, I used to reconstruct the entire constitution of my training school. And "reforming" fits were strongest on those terrible "evenings out" of the wardmaid, and those long Sundays when the washing of mugs and the replenishment of huge ward coal-boxes fell to the lot of the "youngest probationer." Subsequently I have looked back gratefully to the lessons learned in the little ward kitchen where I first discovered that the art of boiling milk without burning was a piece of knowledge not to be despised. My initiation into practical housework began on that morning when I made my début in my first ward—a woman's surgical—and had a gigantic broom put into my appalled hands in a most matter-of-fact way by the staff nurse. In humiliated surprise it flashed across my mind that during my whole existence I had never swept a floor. And here, outstretched before me, was an extent of boarding expanded by terror into an area of acres. And the "staff" looked by no means indulgent. Grasping the broom, I determined to do my best in the unfamiliar industry to which I was called. And I thought the smiles appearing on several countenances must be the outcome of my own self-consciousness until a good-natured convalescent came to my rescue. "Bless me, miss," she said, "that aint no way to 'old a broom." Under her instruction I accomplished the task, and it was a useful revelation that so common a circumstance as sweeping has its subtleties. For many such revelations I have had cause, as probationer, private nurse, and matron, to thankfully acknowledge the wisdom of my training school, which included in the curriculum of the nurses a share in the domesticity of the ward. The elimination of this useful factor from the "new training" is the worst fault of the so-called "progressive policy." But no doubt a compromise will be effected whereby the raw probationer, during the first three months of her training, will be held responsible for a certain amount of dusting, sweeping, and housewifery. It is invaluable to a private nurse to be able to deftly, noiselessly, and quickly lay and light a fire. And it becomes a matter of pride on emergency to perform such a simple little duty—which, alas! some nurses regard as derogatory. I have constantly seen the curious anomaly of a nurse in charge of a very "bad case" who carefully, and with much forethought, strews the hearth with sand, lest a falling cinder or a dropped coal might disturb the restfulness of her patient, showing no hesitancy in admitting a more or less roystering housemaid to "do the grate" with boisterous activity or to "turn out" the room with zealous vigour.

In ninety-nine cases out of a hundred the housemaid can and should be responsible for the cleanliness of the sick-room. But in that hundredth case—when life and death are trembling in the balance, and every nerve in the sick person's body seems raw and sheathless—what an incomparable boon it is if his nurse have the courage and the skill to tide over for a few critical days the tortures of "a room cleaning," by undertaking the duty herself. What a subtle science she may put into the work, as imitating a capital German custom, she lightly wrings a cloth out of water, and fastening this loosely over the hairy portion of the broom, she quietly sweeps the room. The damp cloth absorbs the dust, thus obviating the necessity for sawdust sprinklings. And the subsequent "dusting" will be a small matter, since practically no dust rises to settle. Bare boards or carpets may be equally well swept by this method, though the former have no place in a sick-room unless in infectious illness. In this detail I beg to differ from the conventional book on nursing. Many of these little nursing guide-books are written to pattern, and advice is gravely given to the nurse to "in all cases remove the carpet as the first step on taking charge of a sick person in a private house." In infectious cases this is strictly correct, but in other cases of illness such a proceeding is a grave error of judgment. An uncarpeted room is cheerless, noisy, and incurably draughty. Another traditional point which private nurses would do well to ignore in text-books of this pattern is the insistence that "the ideal sick bed is 3½ feet wide." Teacher after teacher insists on this point, writer after writer dwells upon the excellences of such a bed, till one wonders if such advisers have ever themselves been ill. For most sick persons have experienced the joy and luxury of a bed large enough to toss about in, wide enough for latitude to limbs so weary as to seek all possible changes and variations in position whose very novelty affords relief and rest. In some cases a narrow bed saves the nurse an infinity of weary bending and arm stretching. But in no disease as yet compassed by the complications of our civilisation does any advantage accrue to the patient by condemning him to a narrow bed. And, after all, the patient is a somewhat important consideration in the ethics of illness.

But tradition is a curious factor in life, and takes strong hold unless one has the courage to think things out for oneself. Certainly the "narrow bed" doctrine has been so carefully instilled into the minds of generations of nurses that the belief in their efficacy is almost a matter of heredity. In much the same way the unfortunate custom of drawing cot coverings so tight as to be a terrible discomfort to their small occupants has become traditional. In a walk through the wards of a Children's Hospital, the ingenuity of these small persons to evade the result of their nurses' notions as to order and tidiness is most noticeable. In her ambition to preserve a uniform symmetry in the cots throughout the ward she has fastened the coverings so tightly that a large proportion of her little charges boldly sit on their pillows, keeping only a few inches of pink toe and ankle "under the bedclothes." The constraint of being fastened down by such tight bedding is not unlike that of a "strait-waistcoat," and will prove unpopular in private practice.

The private nurse is wise if she forgets some of the "traditions" of her ward, and consults the friends of her patient as to his general tastes and prejudices, for these may be decidedly strong, and it is worse than useless to run counter to them unless in the patient's real interests. It is often a sore point with the family of a patient to be entirely shut out from nursing councils, and since it is impossible to appeal to them in professional matters it is well to make the most of personal questions, as to which they can often give valuable points.

One of the chief difficulties in private nursing is to adapt the room set apart for the sick person—often a most unsuitable apartment—to his needs, and to overcome such disadvantages is not always an easy art.

Private nursing will be a less complicated calling, if in the houses of the future a model sick-room be included as a part of routine architecture. At present a possible nursery is put on the plan, the needs of the smoker are borne in mind, and a temple is erected wherein the billiard player may devote himself to cue and ball. And there is no logical reason why a room should not be designed for use in time of family illness. Sickness is almost a matter of course, and there is nothing "uncanny" in making provision for its occurrence. I therefore commend to the enterprising architect the idea that he should include in his schemes for delightful homes a model sick-room, sunny, screened from east winds, with noiseless doors, and special fireplace. It should be not too remote from kitchen, and hot water should be laid on, while a small adjoining room wherein all paraphernalia might be neatly disposed of in roomy cupboards would make up an ideal of a little home hospital. What successful cases might possibly be achieved by the doctor, the nurse, and the model sickroom of the future!

### Probationers: Then and Now.

THEN.

She used to come at six o'clock,
Or just in time for tea,
In quiet frock of grey or brown
As neat as neat could be.
Her hair was smoothly plaited up
And guiltless of a fringe,
Her hat with plainest ribbon trimmed
Of sober Quaker tinge.

She brought with her one modest trunk
Containing all she had,
Which did not leave much room inside
For any modern fad.
A stout umbrella, waterproof,
Some gowns that did not fit,
A dozen aprons and some caps
Were mostly in her kit.

She did not have to know a lot,
She never had to "cram,"
To pass, as nurses do to-day,
Some terrible "exam."
Her rules but asked that she should know
Just how to read and write,
And then it was considered, she
Was educated quite.

'Tis true she had to sweep and scour,
And keep the brasses bright,
And wash and iron bandages,
And work from morn till night.
She always came expecting this,
And hoped for nothing more.
For in those days no scrubbers used
To clean the stoves and floor.

She seldom grumbled at the food,
For breakfast was not late;
She always called each student "Sir,"
And wore an air sedate.
She held her staff nurse in respect,
The Sister viewed with awe—
And then the Matron—why, she thought
Her lightest word was law!

She often was a timid girl,
Who had not much to say;
She did not argue every point,
Nor strive to have her way.
She never tried to rule the ward,
She did what she was told;
And somehow managed to get through
Her duties manifold.

Her cubicle was always neat
With decorations few;
Perhaps a book, or china vase,
A photograph or two.
The heels of all the shoes she wore
Were nothing if not low;
She wore no jingling chatelaine
Nor wallet neat; —but now—

NOW.

"Nous avons changé tout cela,"
As everyone may see;
Probationers that come to-day
Aren't what they used to be.
A cab stops, with as many trunks
As any queen might like,
Well loaded up inside and out,
And on its roof—a "bike"!

Its occupant is self-possessed,
She rings the porter's bell,
And calls a sister passing by
To lend a hand as well.
She loads her up with shawls and wraps,
And all her light effects;
And sees that what the porter does
Is just as she directs.

One trunk contains her cycling suits,
The next her tennis dress,
And what with racquet, shoes, and balls,
She could not do with less.
She also brings her golfing clubs,
And even a guitar;
Indeed, we almost might expect
To see a motor car!

She *is* a "new" Probationer,
The newest of the new!
And knows already more than we
In three years ever knew!
Her hair is dressed quite à la mode,
Her dress is up-to-date;
And as we gaze, we wonder what
Will be her final fate.

She goes down to the dining hall,
And takes a vacant chair,
And never moves, although she's told
A staff nurse should be there.
She calls the Sister "Dear old thing,"
But says her cap's a "fright,"
And calls her cubicle a "den"
When going up that night.

When we, who once were new ourselves,
Approached with kind intent
The homesick one—she looked at us,
And wondered what we meant.
Homesick? Not she! She quite allowed
That *we* no doubt felt strange
At first, because we'd not been used
To travel and to change!

Traditions are not in her line,
She laughs at everyone;
She hates conventionalities,
Thinks rules are overdone.
Her cubicle's a sight to see,
With photos, books, and things;
She evidently means to stay,
To see the goods she brings.

She brought a brand-new fancy dress,
In case there is a ball;
Her dancing shoes and op'ra cloak,
Smart blouses—ten in all.
She talks in loud and cheerful tones,
And walks with noisy tread,
And views her future work without
A shade of nervous dread.

But afterwards she settles down,
And when some months are past
We view with fresh surprise, the girl
Who once was "new" and "fast."
If she has sense—she often has—
Her aspect soon will change,
And she will drop the ways that made
Us think her once so strange.

She finds she does not know so much
As once she thought she did;
Concludes it is advisable
To do as she is bid.
And though exams. are passed with ease,
She's willing to admit
That if you can't be practical,
For nursing you're unfit.

The sports that were her heart's delight
Assume their rightful place.
Her hair no longer now attracts
Attention from her face.
Her voice has gained a gentler tone,
Her feet a quiet tread;
Her face is one the patients like
To see beside the bed.

No longer in society
Her company is sought;
They say she's not so brilliant, nor
So gay as once they thought.
Her tennis is indiff'rent, and
They think it's quite too bad
To give one's time entirely to
This novel nursing fad.

She does not think so now, nor sigh
At what she's given up.
She finds it nobler work to help
To sweeten sorrow's cup.
She tries to brighten shadow'd lives
By helpful word and deed
And skilful work that never fails
To meet each passing need.

Her face takes on a diff'rent look,
She's on her work intent,
She takes each duty as it comes,
Nor is on pleasure bent.
And yet she can be "jolly" still,
For, when her work is done,
Off duty who so gay as she,
So bright, and full of fun?

So though our "new" probationer,
Is diff'rent from the old
And orthodox opinions will
Refuse to have and hold;
Although she rides a bicycle,
Plays golf and tennis too,
In spite of all her faults, I think
We'd rather have the "New"!

E. M. F.

### The Nurses' Co-operation.

RESIGNATION OF THE LADY SUPERINTENDENT.

We have to announce the resignation of Miss Phillipa Hicks, lady superintendent of the Nurses' Co-operation. This fact is not only much regretted by the committee and members of the society, but will be felt to be a matter of material loss to medical men and the public who have come in contact with Miss Hicks during her tenure of office. Miss Hicks has been an ideal occupant of her post. Her admirable tact and knowledge of the capacities of her staff, and her invariable courtesy towards those seeking the services of a nurse, are qualifications so valuable that it will render it difficult to find a successor of equal efficiency. The Co-operation has known no other lady superintendent, and it is to her energy and wide connection that the prosperity of the association is largely due. Miss Hicks was trained at St. Thomas's Hospital. She worked at the St. Marylebone Infirmary and the Brompton Hospital for Consumption, and then entered King's College Hospital as night superintendent and ward sister. During the Egyptian Campaign of 1895 Miss Hicks was sent out by Florence Nightingale to nurse the English troops, and on her return she again entered King's College Hospital as home sister. She resigned this post to become matron of the Hospital for Sick Children, Great Ormond Street, where she remained for three years. On her resignation Miss Hicks proposed to retire from institutional work, but her fitness for the post of lady superintendent was so urged upon her by the members of the committee (of which she was one) of the projected Co-operation that she consented to fill the office, in which she has successfully worked for six years. Miss Hicks began work with a staff of 13 nurses, which has been increased steadily until it numbers considerably above 400 at the present time. The progress of the Co-operation during the six and a half years of its existence under Miss Hicks' guidance has been very remarkable. The energy and interest which she has displayed has, no doubt, made this success possible; but, at the same time, it has rendered the thought of rest welcome, and we trust that Miss Hicks will enjoy her well-earned leisure and the best of health. She will leave behind many regrets, and carry with her the best of wishes of her fellow-workers, her staff, and her friends.

Miss Blanche Trew, who has acted as assistant matron to Miss Hicks for two and a half years, has resigned her appointment. Miss Trew was trained at University College Hospital, afterwarde becoming sister at the Derby Royal Infirmary. She worked on the staff of the Co-operation for two years before undertaking her present post, and intends returning to nursing work again in connection with the association. Miss Trew has throughout her tenure of office been a most indefatigable and loyal worker, and we are glad to learn that the Co-operation will not lose her services.

### Where to Go.

National Health Society.—Lady George Hamilton will present the diplomas, medals, and certificates of the society to successful candidates at Grosvenor House, on Saturday, May 22nd, at 3 p.m.

Royal United Service Institution, Whitehall.—The annual meeting of the Children's Country Holidays Fund will be held on Monday, May 24th, at half-past three. The Earl of Erroll will preside.

The Maternity Charity and District Nurses' Home, Plaistow, E.—A reception by the committee of this charity will be held at the Town Hall, Stratford, on Tuesday, June 1st, from three to six p.m., at which the Mayor of West Ham has consented to preside.

Wembley Park.—Nurses may be glad to know that the Sunday sacred concerts at Wembley Park have proved such a success that they are being continued through the summer. The afternoon performance begins at 3.30, and the evening one is given in the large hall at 7 o'clock. The public are invited free to the concerts, which will doubtless add much to the already great attractions of this pretty place.

Victorian Era Exhibition.—On Monday this exhibition will be opened at Earl's Court by the Duke of Cambridge. Nurses will be interested to hear that the Scientific Press has secured a stall in the Nursing Section, which they hope to render both attractive and useful. A comprehensive selection of nursing literature and other exhibits appertaining to the work of nurses will be here on view.

### The Hospital for Sick Children.

A new home for the private nursing staff of the Hospital for Sick Children, Great Ormond Street, was opened on Wednesday, when a large number of visitors and members of the hospital staff attended and inspected the place. The home contains a dining-room, a sitting-room, and a usefully-fitted little laundry. A room is set apart for the sister in charge, and there is at present sleeping accommodation for ten nurses. The conduct of the place is under the supervision of Miss Smedley, matron of the hospital.

### Nursing in Egypt.

A nurse's work in Egypt is quite unlike anything in England. If she is a private nurse her cases will be chiefly in the hotels, where she has no nursing appliances, or conveniences, where she will be devoured by mosquitos, and agitated by the "suffright" (native boy who does the housework). If her work lies in hospital, her sense of "love for the beautiful" will suffer much, and that, perhaps, will be the least of her trials. There are several hospitals in Egypt worked by English nurses. Some years ago (it might be 14 or 16 years since) Lady Strangford started hospitals in the principal towns. That in Cairo was a great success for a time, and used to a large extent during the Nile expedition of '82-84; but eventually had to be closed for want of funds, and the head nurse set up as a laundress, worked hard, and struggled on till the beginning of this year, when she had to relinquish even that "profession," as it did not pay.

Just before the Port Said Hospital was opened Lady Strangford died. It was the last hospital, and, oddly enough, the only one out of all those started which now exists. It is a nice little place, built in Bungalow style, and near the sea. There is a resident house surgeon, a sister, and three nurses. The patients are principally passengers and sailors from the ships passing through the Suez Canal who are taken ill and have to be landed. The nurses have plenty of work to do, and are very happy, but Port Said is not much of a place. The climate is hot and damp, there are very few English residents, the inhabitants are chiefly Arabs, Greeks, Maltese, Indians, Cingalese, and all of the lowest class. The town is very ugly, there are no trees or gardens, nor, as far as one can see, any of the little pleasantnesses that go to make up the great aggregate of life.

In Cairo there is the English Government Hospital in the citadel solely for the British soldier, and nursed by the army nursing sisters, with their pretty grey uniforms and red capes. This hospital is part of a palace which formerly belonged to the Khedives of Egypt. It is an enormous building, with large wards, which have elaborately-painted walls and ceilings, and boasts an Alabaster staircase.

Next comes Kars-il-Aini, the Egyptian Government Hospital, which contains about 500 beds, and is for native patients only. When Dr. Milton took over the management of this hospital some years ago he introduced English nurses, and for some time found it uphill work getting anything like system established. There was little accommodation for nurses of any kind; at first they fell ill, one died, and it was some time before things could be brought into a satisfactory state. At first the Khedive would only consent to the nurses nursing the women, but later on they were allowed to nurse in the male wards too. They are now rebuilding the hospital, and as far as possible on the plan of St. Thomas's in London. There is a medical school attached to the hospital and a nicely built nurses' home in the grounds, where the six English nurses engaged in the hospital reside. There are a great many native girls being trained as nurses; these, however, only work in the women's wards, and always veil their faces when the students go round. They are most lackadaisical, graceful girls, childish in mind, and amusing to watch. The nurses at Kars-il-Aini see a lot of good work, and lead a very busy life. If some of those nurses at home who make an idol of their wards could but peep into the native wards, the shock to their feelings would be great! Imagine a long ward with bare whitewashed walls, wooden ceiling, and stone floor, a row of low iron eds on either side, and a deal table in the centre, and you have the native ward; no luxuries or flowers, or anything of that kind are to be seen. The native has not been educated in that direction, and is in clover if he can only get himself rolled up in a blanket and be allowed to lie on the floor. The windows are nearly always open, and the insolent little sparrows enter in the most fearless manner, fly about, pick up food, and even build in the wards. There is not much ceremony about feeding the native patients. When dinner-time arrives a large sheet of oilcloth is spread on the floor, an enormous pewter tray is placed in the centre, on which is placed the food—usually boiled mutton and rice or beans and stewed chickens (bony ones). Then the patients who are up—having first washed and prayed—sit on the floor round the dish, and each with a round cake of native bread attacks the contents. Knives, forks, or spoons are neither provided nor needed; fingers and bread seem to be quite sufficient. An enormous tin can of water is now passed round, each person takes a long draught, and the meal is over. To clear away is a small matter—remove the empty tray, roll up the oilcloth mat, and the thing is done. It is quite simple, and a great saving of labour.

Nursing outside the hospitals is somewhat erratic. Until 1890 there were only three or four private nurses for the whole of Egypt, and, with the tremendous number of travellers and tourists visiting the country, it can be seen how inadequate this number was and how the tourists who fell ill suffered in consequence for want of proper nursing. In 1891 Miss Helen Thomson opened a private nursing home in Cairo, got out trained nurses from England, and so provided for a certain proportion of the sick travellers. And soon after that the proprietors of the Mena House Hotel at the Pyramids thought it advisable to engage a nurse exclusively for their visitors. The hotels at Luxor and Assonan, Upper Egypt, followed the example. In addition to this a certain number of nurses find their way out to Cairo each year in the hope of finding private work. So that in one way and another there are quite a number of them nowadays, and each year lately the number increases, and many invalids travel with their own nurse.

All the hotel nurses and most of the private nurses are obliged to return to England at the close of the season, as there is then no work, living is expensive, and the climate during the summer trying.

There is an opening for a central home in Cairo for the unattached private nurse, and perhaps some one with the experience and money necessary to start such a thing may feel inclined to do so ere long.

### A Day of Rest for Nurses.

In the summer of last year an opportunity was provided for those London nurses who were able to avail themselves of it to spend a "quiet day" at Hertingfordbury. Three short services were arranged at intervals during the day, allowing the nurses full opportunity for enjoying the country and hospitality offered to them, so that the actual expense to each nurse did not exceed a few shillings, being the cost of the return fare from King's Cross. A similar invitation is now given for Thursday, June 3rd, when the Bishop of Wakefield has kindly promised to give the address at each service. Any information as to details may be obtained from the secretary of the Hospital Chaplains' Union, the Rev. A. G. Locke, St. George's Hospital, who is kindly acting officially in carrying out the arrangements, or communications may be made to Canon Burnside, Rector of Hertingfordbury, Hertford.

### Can the Illiterate and Unphilosophical Rise Superior to the Things that Surround Them?

By a Modern Nurse.

Circumstances, or the things that surround us, are frequently spoken of as though they were so many forces putting in motion our lives' deeds, and actions, shaping our conduct and general mannerism, moulding and influencing our thoughts and views, maturing our minds, developing our intellects, and even making our destinies for us, as though Nature had decreed from the beginning that we ourselves—we, the most concerned—should have neither hand nor voice in the glorious work of so transcendental an undertaking. Moreover, these "so many forces" have come to be looked upon in the light of so many dead but active creatures that partake of the character of the things that produce them; like the statical and dynamical forces in mechanics which, whatever be their nature, and despite certain resistance, produce motion and tendencies to motion in a material body or bodies. Whether, for example, the dynamical force be represented by the quick, powerful blow of the cricket bat that puts the ball in motion, driving it in any direction, or whether it be as the quiet pressure of the potter's mould upon the tempered clay, forcing it into some other form, we cannot fail to observe that in opposition to some amount of resistance, namely, that of the colliding ball and that also of the material clay, the position and direction of the first and the shape and worth of the second, are evidently altered after the influences or forces produced by the things that were brought into immediate contact with them. This alteration did not previously exist. It is the force of circumstances that makes all the difference; and while inanimate objects or bodies are thus influenced and altered, so, too, are the physical and mental conditions of human beings, only not so quickly.

But, while it is to be noted that there are inanimate bodies that have some real resistance, as though they possessed being, and, notwithstanding there are among human creatures those who live actively, exert themselves, are morally trained and thoughtful, using their will with regard to the things that surround them, and determined not to "let things slide," nor allowing even the influences to come and go without an intelligent consultation, yet there are also those among human beings who are so passive and inert in their cold indifference to the things and influences that surround them as to impress one as only "the dead" can. If they were as lifeless as the inanimate things that make and limit their world they could not be more hopelessly lost to the higher influences of the upper world of thought. They are the ball without its resistance driven anywhere; they are the badly-tempered clay spoiled in the moulding, the clay of which the potter may make what he chooses, or the tempered clay, so to speak, that is moulded on the ugly pattern of that wretched, unhealthy, slum surroundings. If it be true that the face is the mirror of the soul—and we need not be very metaphysical or philosophical to understand that and all that it teaches us—we have only to take a walk through the slum districts of any of our large centres of population and quietly study human faces as we pass along; we shall soon perceive that these faces are almost destitute of animation and of every line of intelligence save that which is marred by its very sensuousness—expressions indicative of mingled hardness and yielding weakness, brutal passion and passive indifference, that can only produce the conclusion that these unfortunate creatures who are there for the most part, and so moulded, owing to the force and power of antecedent circumstances, of which they are the results, are wretched and miserable, but do not realise it. They seem as ignorant of their state as their dogs are of eternity. Truly, ignorance would be bliss if it were folly to be wise. In this connection then it may be observed that it is not in Central Africa but among those physically, mentally, morally, poverty-stricken creatures here at home, in Central Slumdom, where the labours of love are truly needed—the philanthropist, the schoolmaster, and the clergyman, these three of whom the greatest is neither, for they are equally necessary, co-ordinate, their great object being the improvement and ennobling of human beings in body, soul, and mind This is St. Paul's definition of a man—"body, soul, and spirit"—and this is the view, I am glad to observe, that the Church, more than ever she did in the past, is taking of humanity at large in regard to her work amongst the wretched and unfortunate. Speaking for myself, I am strong in the belief (and personal observation confirms me in it) that it is only the morally and intellectually educated who rise superior to their surroundings. The illiterate and unphilosophical, such as I have described them, never rise, but do so to fail ignominiously, even living and dying where or as they were born and "dragged up," without knowledge of themselves or their surroundings, and never seeking to know how they might rise superior to the gutter or the slums.

At the head of this paper stands the question, "Can the illiterate and unphilosophical rise superior to the things that surround them?" And it has been shown that the forces of circumstances are not only so powerfully set against their ever accomplishing it, but also of their even attempting so much. It must strike every thoughtful individual that, unaided by religion, the clergy, and the schoolmaster, and uncared for by philanthropy that will give healthy employment and insist on the cultivation of habits of thrift, these people will ever continue to wallow in the mire—poor, wretched, and lost, illiterate and unphilosophical, without any idea or incentive to attempt the scaling of the gloomy, shady heights that bound their wretched mould.

If there were no adverse powers to overcome, and if going with or resisting any influence made no difference in us or our surroundings, we should not need to be forewarned or equipped in early life for life's battles, for there would be no battles to fight.

It is, however, easy to float with the tide, and not difficult to sail with the wind; but a combination of powers that will resist both wind and tide is better represented by the modern high-class steamboat ploughing the deep against strong head winds and heavy seas than by the noble ship, under close-reefed topsails, plunging and beating about in the storm as she labours, perhaps in vain, to enter the harbour. It is so with individuals—some failing, others succeeding; and the great difference between success and failure, which is ofttimes ascribed to those "circumstances which alter cases," may only be regarded as a mere matter of accident, or of the luck that attends some people in the "smartness" with which they avail themselves of time and of the opportunity when it offers. Still, it must be admitted that many individuals have by their intelligence and ability rendered their surroundings favourable to their attaining to success.

In view of what has already been observed, the best and richest heritage which parents can bestow upon their children is the "combination of powers," if I may so speak, of a good sound moral and intellectual training; in other words, an education in which the moral and intellectual have been so carefully blended and interwoven as to leave no open door for the least possible chance of the moral ever being divorced from the intellectual, and *vice versa*. It is this power above all others that has ennobled thousands of people in every age, making them infinitely superior to self and surroundings. It was this power that made the great Apostle to the Gentiles superior to fanaticism, to the bigotry that causes enmity and bloodshed, and superior, too, to selfishful assurance and blinded zeal. And nothing less than this power can make man superior to the transient things of time, especially superior to those things whose adverse and evil influences will ever keep him "a little lower than the angels."

### Minor Appointments.

Hartlepool Nursing Guild.—Miss Jessie Leavis has been appointed District Nurse to this institution. Miss Leavis was trained in the Belfast Royal Hospital, afterwards working on the private staff of that institution. Her testimonials are excellent.

### The Prince of Wales at Oxford.

### THE SARAH ACLAND HOME.

The visit of the Prince of Wales to Oxford was made the occasion of much festivity and rejoicing, and His Royal Highness must have been gratified by the enthusiastic welcome he received. To nurses the most interesting event of the visit was the opening of the new buildings of the Sarah Acland Nursing Home and the unveiling of the tablet which briefly records the history of the institution and which runs as follows:—

"To perpetuate the revered memory of Sarah Acland a home for district nurses was established in Oxford by her friends, A.D. 1878. This home was afterwards enlarged so as to receive private patients and a staff of private nurses. Her husband, Sir Henry W. Acland, Bart., K.C.B., retired from the Regius Professorship of Medicine in 1895, having held that office for nearly forty years, and his many friends agreed to record by a testimonial their sense of the services he had rendered to the University, the City, and the medical profession. By his desire this testimonial was devoted to the development of the medical care of the sick by the erection of this building, which was opened by H.R.H. the Prince of Wales, May 12, 1897."

Mrs. Liddell, as president of the committee, received the Prince of Wales on his arrival at the home, and in her address stated that the new buildings were an outcome of the sum of £3,000, subscribed by his friends to Sir Henry Acland as a testimony of esteem, and of a further sum of £1,000, subscribed in memoriam of Mrs. Acland's nephew, Mr. H. B. Cotton, by Mr. R. Lehmann. The scheme, which started with the employment of one district nurse, has so developed that five district nurses are now at work, a staff of 25 private nurses is maintained, and 61 private cases during the past year have been treated in the home. The provision of a Home where private patients can be received has proved very valuable in Oxford, where there are many undergraduates who, when taken ill, are inconveniently housed in college rooms or in lodgings. A bed for poor gentlewomen has been endowed at the home by the Master of Pembroke College in memory of his daughter Rose.

The Prince of Wales, replying to Mrs. Liddell's address, said: Nothing could have given me greater pleasure than to take part in this interesting ceremony, first of all, because of my old Oxford days and the friends I made here, among whom I number my valued friend the Dean and Mrs. Liddell, and many who were my undergraduate friends; and for the sake of Mrs. Acland and her kind and excellent husband, Sir Henry, I am glad to think that in memory of the one and in recognition of the valued services of the other this home has been established. I cannot conceive anything more useful and meritorious than having a home for nurses, and I am glad to think that you will also receive patients. I sincerely wish this institution all possible success, and that it may grow bigger and bigger every year. His Royal Highness then declared the home open. A large number of visitors were present, amongst whom was Sir Henry Acland, unable, to the regret of all, to address the assembly himself owing to the feeble condition of his health. His son, Captain Acland, said a few appropriate words for him, thanking the Prince for his services to the institution. The old Home has long proved insufficient to meet the demands upon it, and it is besides not very conveniently arranged. The new premises will provide all that is required. The Sarah Acland Home is now established on an excellent and permanent basis, such as would have rejoiced the heart of the good and gentle lady in whose honour it was founded, and it will help to perpetuate the memory of her husband, one of the most respected members of Oxford University, who was ever associated with her in all works for the good of humanity.

### Everybody's Opinion.

[Correspondence on all subjects is invited, but we cannot in any way be responsible for the opinions expressed by our correspondents. No communication can be entertained if the name and address of the correspondent is not given, or unless one side of the paper only is written on.]

### NURSES' UNIFORM.

"A Would-be Member" writes: I have read with much interest the suggestion of having a home for nurses in their old age. The same idea has occurred to me frequently, as I have come across many nurses who dread the lonely life in the future. I think such a "home" would be the greatest boon and blessing to many a lonely soul. Therefore I am sure the suggestion of a guild would be warmly welcomed and well supported, but I think it would be wiser to have it started by a body of able gentlemen and a committee of nurses. If a guinea a year was the subscription I feel sure we would have hundreds of members, and many would gladly give this small amount to help their fellow sister. The letter from "A. B. M." is practical and well worth consideration.

### NURSES' UNIFORMS.

"Nurse H." writes: It strikes me as a little amusing that "A Sister" should be the one to advocate a-coat-and-skirt-and-sailor-hat "uniform." Here is the new woman idea and no mistake! For what does the very name of "sister" suggest but the cloak and bonnet and veil of the "religious" orders, who ever since earliest times have devoted themselves to nursing, and whose nineteenth-century followers I suppose we are? "Queen's Nurse" makes a capital suggestion in answer to my letter, viz., "to get up a petition praying for the protection of our uniforms"; not so much though, it seems to me, is it necessary to protect them from poor little nurse girls, or from any who are earning a honest living, as alas! from those who are not. And this should be set about at once and in earnest. A petition, signed by all the Royal National Pension Fund nurses, might be thought of.

### DISPENSING.

Miss B. E. Thompson, dispenser, Birmingham and Midland Hospital for Women, writes *re* "Dispensing": As my letter has evidently been misread, allow me to say I always prefer that my pupils should have passed the preliminary Pharmaceutical examination before becoming pupils in the dispensary of the Women's Hospital. Then they are free to go on with the practical work for the Apothecaries' Hall and the minor examination. In the rules *re* candidates as pupils, it stated that "preference is given to those who have passed either the preliminary or any equivalent examination." I think it would be a mistake to ignore this first step, and then for the girls to find they were obliged, after several years of practical work, to go back to their Latin grammar. My pupils have done exceedingly well and hold good appointments, and after fourteen years' experience I consider it is a good plan to be as particular with ladies as one would be with youths. Why should girls get into the work of pharmacy more easily than boys?

### ONE-SIDED CHARITY.

A Correspondent writes: It has surprised me that there has been comparatively so little open criticism of the part taken by a certain section of people in this country in the matter of supplying medical and nursing aid to the Greeks wounded in the present war. One is, of course, naturally unwilling to carp at any action taken in the name of humanity and philanthropy, but to the initiated the *Daily Chronicle* National (?) Fund tells more of a political movement than anything else. The whole manner in which medical aid has been sent out to the seat of war has been surely misguided. If England as a country wished to do as she has always been ready to do, succour sick and wounded men in time of war, why was this not done through the Red Cross Society, and aid placed equally at the service of the Greeks and Turks? Surely the strictest impartiality should be preserved, and help meted out to both sides alike, or as a nation we are not maintaining a neutral attitude, but giving very material assistance to one country against another. One cannot but see that self-advertisement and party feeling have largely been responsible for the movement which has resulted in a certain number of people in England actually paying over twenty trained nurses two guineas a week to undertake the whole organisation of the ambulance department of the Greek army, itself absolutely unequipped in this particular. I am glad to see that even thus late in the day the English Red Cross Society has offered aid to the Porte, but it is a grievous pity that the whole movement was not under its auspices, and the assistance sent impartially to the wounded of both armies.

### For Reading to the Sick.

COURAGE.

"THE hidden Force that makes a Lifetime strong."—*Lowell.*

**Verses.**

Breast the wave, Christian,
When it is strongest;
Watch for day, Christian,
When the night's longest:
Onward and onward still
Be thine endeavour;
Seek the rest that remains
To thee for ever.

Fight the fight, Christian,
Jesus is o'er thee;
Run the race, Christian,
Heaven before thee:
He who hath promised it
Faltereth never;
He who hath loved so well
Loveth for ever. —*Joseph Stammers.*

Soldiers of Christ! arise,
And put your armour on,
Strong in the strength which God supplies
Through His eternal Son!
Strong in the Lord of Hosts,
And in His mighty power!—
Who in the strength of Jesus trusts
Is more than conqueror.
From strength to strength, go on!
Wrestle and fight and pray!
Tread all the powers of darkness down,
And win the well-fought day! —*Wesley.*

**Reading.**

Strong always to satisfy even when they cannot save.—*Ruskin.*

Be strong and of good courage, that thou mayest have good success whithersoever thou goest.—*Joshua* i. 7.

We make our victory a great deal more difficult than it ought to be by want of courage. There are many faults and many weaknesses which require nothing more than a decisive effort, a determined push to overcome them at once and for ever. If you want to live a Christian life, do not dally with your purpose; do not fancy that you will find it easier to win your way by degrees, and that by a gradual change you may attain to the same end with less pain than you fear will be given by a sudden wrench. Nothing can be a greater mistake. Press into the enemy's citadel at once; do not wait outside till he has had time to shoot you down. . . . Be assured that God will help you. Be assured that Christ will give you strength. . . . I know you will meet with many failures between this and the grave; but I am sure that you will meet with fewer failures in proportion to your courage, for this kind of courage is but another form of faith, and faith can work any miracle whatever, even the greatest miracle of all, bringing your soul to God.—*Bishop Temple.*

Fallen threads I will not search for—I will weave.—*G. Macdonald.*

### Notes and Queries.

*A Mistake Somewhere.*

(256) Can you inform me and the public if it is customary in private nursing institutions to have a rule "that every nurse has to report to the matron everything that takes place in the house" where she may be nursing a case? I have had to do with nurses who have stated that they reported "everything to their matron according to their rules."—*Surgeon.*

There must be some misunderstanding somewhere. The matron of a private nursing institution naturally requires her nurses to report to her concerning their work, and anything which may affect their work, but one of the first and most important precepts instilled into private nurses is the absolute necessity for their treating their patients' private concerns with the utmost discretion, and the avoidance of anything approaching gossip respecting the private and domestic affairs of their patients.

*National Home Reading Union.*

(257) Please tell me the address of the above society in London?—*C. J. R.*

The address is Surrey House, Victoria Embankment, W.C. Miss M. C. Monday is the secretary.

*Epilepsy.*

(258) Can anyone recommend a home for a girl aged nine who suffers from epileptic fits? Parents could pay 6s. a week.—*Mrs. P.*

Write to the Meath Home of Comfort for Epileptics, Westbrook, Godalming, or to the National Hospital for Epilepsy and Paralysis, Queen Square, W.C.

*Massage.*

(259) Will you kindly let me know where I can go for a course of massage to gain a certificate? I want the very best. I am a fully-trained nurse.—*A. R.*

Write to the secretary of the Society of Trained Masseuses, 12, Buckingham Street, Strand. You cannot do better than obtain the certificate of this society.

*Training.*

(260) I am very anxious to train as a nurse, and have answered several advertisements for probationers in your columns, but have had no replies. Please advise me what to do? I am 21, healthy, and well educated. Is there any charge for answering queries, please?—*Dunelm.*

You are too young for most general hospitals, and had better apply to the matrons of some of the children's hospitals where probationers are admitted from 19 or 20. In "How to Become a Nurse" (Scientific Press, 28 and 29, Southampton Street, Strand, London, W.C.) you will find the names and addresses of the various hospitals, with the particulars of the training and the age at which probationers are eligible. There is no charge for answering queries.

*Children's Nurses.*

(261) Please tell me the address of the institution in London where nurses are trained for the care of little children and in general nursery work?—*Mrs. Coope.*

The Norland Institute, 29, Holland Park Road Avenue, W.

*Dispensing.*

(262) Can you tell me where is the best place in London for a lady to learn dispensing thoroughly and quickly?—*E.*

Read the article on "Dispensing" in THE HOSPITAL for April 3rd of this year. Pupils are taken by the lady dispensers at the New Hospital for Women, Euston Road, N.W., and at the Temperance Hospital, Hampstead Road, N. You had better apply to one of these ladies.

*Guild of St. Barnabas.*

(263) Please tell me how I can join the guild of St. Barnabas for Nurses?—*A. E., Mon.*

Miss Wood, 27, Percy Street, Tottenham Court Road, W., is the London Secretary for the guild, and you had better write to her for particulars. There are branches of the guild all over the country, and you may like to join the nearest, which in your case would be Gloucester or Bristol. Get the monthly paper of the guild, "Misericordia," published by W. Knott, 26, Brooke Street, Holborn, E.C., price 2d.

*A Nurse's Grievance.*

(264) Please tell me if you think a matron in a poor law infirmary can come and take stock of linen, &c., with only an hour's notice to the nurse in charge, who has several bad cases and no help?—*Infirmary Nurse.*

It is quite impossible to give any opinion on such a matter without being in possession of the whole circumstances of the case. While nursing in workhouses remains in its present state nurses must put up with many inconveniences unknown in hospital work, and, as a general rule, it is better, as far as possible, for the trained nurse to avoid friction with the untrained officials, even if in order to do so she has to put her pride in her pocket and submit to a certain amount of inconvenience.

*Dispensing.*

(265) I am a male nurse, with the Medico-Psychological nursing certificate, and have been working for the Male Nurses' Temperance Co-operation. I contracted typhoid some time ago and have been told I must give up nursing, and now I am anxious to learn dispensing. I am 31, and fairly well educated. Please tell me what steps to take?—*Ipswich.*

Write for advice to the Secretary of the Pharmaceutical Society, 17, Bloomsbury Square, W.C.

*Incurable Home.*

"B. F." asks about a suitable hospital for an incurable consumptive patient. We shall be happy to answer the question if the writer will send full name (not for publication) and will also state whether any payment can be made or if it is a destitute case.

